# Neurobiology of psilocybin: a comprehensive overview and comparative analysis of experimental models

**DOI:** 10.3389/fnsys.2025.1585367

**Published:** 2025-08-05

**Authors:** Dotun Adeyinka, Dayna Forsyth, Suzanne Currie, Nicoletta Faraone

**Affiliations:** ^1^Department of Chemistry, Acadia University, Wolfville, NS, Canada; ^2^Department of Biology, Acadia University, Wolfville, NS, Canada; ^3^Department of Biology, University of British Columbia, Kelowna, BC, Canada

**Keywords:** psilocybin, psilocin, psychedelics, serotonin, animal models, translational neuroscience, therapeutics

## Abstract

Psilocybin, a compound found in *Psilocybe* mushrooms, is emerging as a promising treatment for neurodegenerative and psychiatric disorders, including major depressive disorder. Its potential therapeutic effects stem from promoting neuroprotection, neurogenesis, and neuroplasticity, key factors in brain health. Psilocybin could help combat mild neurodegeneration by increasing synaptic density and supporting neuronal growth. With low risk for addiction and adverse effects, it presents a safe option for long-term use, setting it apart from traditional treatments. Despite their relatively simpler neuronal networks, studies using animal models, such as *Drosophila* and fish, have provided essential insights on the efficacy and mechanism of action of psilocybin. These models provide foundational information that guides more focused investigations, facilitating high-throughput screening, enabling researchers to quickly explore the compound’s effects on neural development, behavior, and underlying genetic pathways. While mammalian models are indispensable for comprehensive studies on psilocybin’s pharmacokinetics and its nuanced interactions within the complex nervous systems, small non-mammalian models remain valuable for identifying promising targets and mechanisms at early research stages. Together, these animal systems offer a complementary approach to drive rapid hypothesis generation to refine our understanding of psilocybin as a candidate for not only halting but potentially reversing neurodegenerative processes. This integrative strategy highlights the transformative potential of psilocybin in addressing neurodegenerative disorders, leveraging both small and mammalian models to achieve translational research success.

## 1 Introduction

In 1957, a compound known as psilocybin was isolated from the *Psilocybe mexicana* mushroom by Swiss scientist Albert Hofmann (Hofmann et al., 1958). The following year, a synthetic version of this compound was developed and has since been used for various purposes, including recreational activities and spiritual or religious practices (Daniel and Haberman, 2017; Lowe et al., 2021; Passie et al., 2002; Teixeira et al., 2022; Ziff et al., 2022), promoting the overall wellbeing of both body and mind (Teixeira et al., 2022).

Psilocybin has been instrumental in managing mental health issues like depression, autism, anxiety, schizophrenia, bipolar disorder, and substance addiction, (Kisely et al., 2023; Ries et al., 2017) all of which are major global challenges (Rehm and Shield, 2019). It was estimated that mental health or substance disorders impacted as many as 1 billion people as of 2020 (World Health Organization, 2020). As mental health disorders become more widespread, especially as a result of the impact of COVID-19, psilocybin-assisted therapies might further help address some of the limitations of traditional psychiatric treatments, such as opioid analgesics (Lowe et al., 2021). Beyond its potential to treat mood and anxiety disorders, psilocybin has shown pain-relieving effects, as observed in several clinical studies on treating cluster headaches (Lowe et al., 2021; Schindler et al., 2015; Sewell et al., 2006) and persistent pain (Whelan and Johnson, 2018).

To date, there is limited research on psilocybin, largely due to the Controlled Substances Act of 1970 (Ortiz and Preuss, 2024), which nearly stopped active research on hallucinogens and psychedelics. Earlier studies were often dismissed due to their small scale and outdated methods, which eventually led to limited funding accessibility for further pursuing this research. Despite all these challenges, interest in its potential medical uses continued. Although research was challenging due to their Schedule I status of psychedelics, progress was made in 1992 when the National Institute on Drug Abuse and the FDA advisory committee allowed psychedelic research to resume (Daniel and Haberman, 2017; Nichols, 2014), slowly re-entering the framework of contemporary science and numerous clinical research (Lowe et al., 2021).

This review examines the neurobiology of psilocybin, focusing on its mechanisms of action and its potential role in the treatment of neurodegenerative conditions and the management of major depressive disorders. We critically evaluate the use of several animal models, highlighting their contributions to understanding the effects of psilocybin on neural plasticity and neuroprotection. By integrating insights from small animal models, such as *Drosophila* and fish, and leveraging the detailed investigations afforded by mammalian systems, we highlight the complementary roles these models play in translational research and the determination of mechanisms of action. Ultimately, this review advocates for the transformative potential of psilocybin in treating neurodegenerative disorders.

## 2 Serotonin (5-HT): the brain’s pathway to resilience

### 2.1 Evolutionary and functional roles of serotonin across animal models

Serotonin (5-hydroxytryptamine) is a key neurotransmitter in the central nervous system and is found in brain regions across many species. Serotonin-producing neurons have been discovered in animals from all major groups, including cnidarians, flatworms, annelids, molluscs, nematodes, arthropods, echinoderms, tunicates, and vertebrates. This suggests that serotonin emerged early in animal evolution (Lillesaar, 2011). In mammals, serotonin assumes specialized functions due to the development of advanced brain structures, such as the neocortex and the complex connections between the cortex and other brain areas (Puglisi-Allegra and Andolina, 2015). Animal studies indicate that the serotonergic hormonal system plays a crucial role in coping with changes in homeostasis as well as neurodegenerative diseases such as Alzheimer’s disease by influencing learning and memory functions. In this disease, it appears that there is specific neurodegeneration in serotonin receptor pathways and decreased activity at serotonergic synapses (Garcia-Romeu et al., 2022).

### 2.2 Psilocybin and serotonin-mediated stress regulation in model organisms

Serotonin (5-HT) plays a crucial role in regulating stress responses in mammals (Hesselgrave et al., 2021). It modulates mood, anxiety, and emotional processing through its action on various receptors in the brain, particularly within regions such as the prefrontal cortex and amygdala (Barrett et al., 2020b; Carhart-Harris et al., 2016; Grimm et al., 2018). Psilocybin, as a serotonin receptor agonist, primarily targets the 5-HT2A receptor, inducing neuroplastic changes that may alleviate the effects of chronic stress, such as hippocampal atrophy and reduced neuronal connectivity. By restoring synaptic function and promoting neurogenesis, psilocybin shows potential in mitigating stress-related neurodegenerative disorders (Carhart-Harris et al., 2017; Castrén and Antila, 2017). We will discuss this in more detail below.

In *Drosophila*, serotonin is equally vital for managing stress and regulating behaviors such as aggression, courtship, and locomotion (Alekseyenko et al., 2014). Psilocybin’s influence on serotonin pathways in the fly brain offers a simplified model for studying its effects on neuronal circuits and stress resilience. The genetic tractability and rapid life cycle of *Drosophila* make it a valuable tool for high-throughput analyses (Arbeitman et al., 2002; Bülow et al., 2020; Rubin, 1988), revealing foundational insights into serotonin-mediated stress responses. Serotonin’s central role in modulating mood and stress responses mirrors its function in mammals (Ries et al., 2017). Recent studies in *Drosophila* have shown that stress-induced changes in serotonin levels can influence various behaviors, including sleep, aggression, and social interactions (Ries et al., 2017). For example, serotonin levels affect the fly’s resilience to stress and its ability to adapt to challenging environments (Ries et al., 2017). When exposed to prolonged stress via repeated exposure to intense mechanical vibrations (300 Hz) for approximately 10 h per day, *Drosophila* display altered locomotion and reduced exploratory behavior, both of which are influenced by serotonin regulation in the brain (Ries et al., 2017). These responses are part of a complex coping mechanism, with serotonin pathways modulating behaviors that help *Drosophila* to conserve energy and survive under stress. Further research on the role of serotonin in *Drosophila* stress responses would provide valuable insights into understanding the molecular and genetic underpinnings of stress resilience and coping mechanisms, with implications for broader neurological studies.

In fish (e.g., zebrafish, *Danio rerio*), serotonin also governs stress and anxiety-like behaviors (Puglisi-Allegra and Andolina, 2015; Soares et al., 2018), with psilocybin showing promise in modulating these processes. Zebrafish larvae are particularly valuable research tools due to their transparent bodies, which enable researchers to observe brain activity directly in real time using imaging techniques such as fluorescence microscopy. However, this transparency is lost as they develop into adults, due to increased pigmentation that obscures internal structures, including the brain. To address this, researchers often use specialized techniques such as tissue-clearing methods or employ genetically modified strains like the “casper” zebrafish, which remain transparent throughout life and enable long-term *in vivo* brain studies (White et al., 2008).

The transparent and accessible zebrafish brain now facilitates *in vivo* imaging of neural activity, allowing for real-time observation of psilocybin’s impact on serotonin-driven circuits (Braun et al., 2024), which has been instrumental in uncovering the molecular and behavioral effects of psilocybin. For instance, studies have shown that psilocybin enhances spontaneous exploration and mitigates stress-induced behavioral disruptions in larval zebrafish (Braun et al., 2024). Neural activity imaging in these studies revealed that psilocybin suppresses the activity of serotonergic neurons in the dorsal raphe nucleus, a key area involved in mood regulation (Braun et al., 2024). These findings suggest that psilocybin induces a unique brain state that is both stimulatory and anxiolytic, providing valuable insights into its potential therapeutic effects.

This research bridges the gap between small invertebrate models and mammalian research for a deeper understanding of psilocybin’s neurobiological potential (Braun et al., 2024). By leveraging the genetic tools and high-throughput screening capabilities of zebrafish, researchers can identify key molecular pathways and connect findings from simpler models like *Drosophila* to the more complex neural dynamics observed in mammals.

## 3 Mechanism of action

### 3.1 Metabolic conversion and cross-species pharmacokinetics of psilocybin

Psilocybin is a prodrug; it requires metabolic conversion in the body to become pharmacologically active. Once ingested, psilocybin is rapidly dephosphorylated by alkaline phosphatase enzymes to form psilocin, which is the active compound that exerts psychoactive effects by binding primarily to 5-HT receptors in the brain (Hasler et al., 1997; Passie et al., 2002).

In rodent models (i.e., rat, mouse), this metabolic conversion from psilocybin to psilocin has been well-documented. These alkaline phosphatase enzymes in the liver and gut catalyze this transformation, and psilocin is then distributed to the brain, where it modulates neurotransmission. Rodents also show dose-dependent behavioral and neurochemical responses to psilocybin and psilocin, making them suitable for studying psilocybin’s effects on neuroplasticity and mood regulation (Halberstadt and Geyer, 2011; Nichols, 2020).

In fish models, such as the zebrafish, the enzymatic pathways for drug metabolism are similar to those in mammals in many aspects. While direct studies on psilocybin metabolism in fish are lacking, zebrafish express phosphatases and other relevant enzymes that could feasibly support the conversion of psilocybin to psilocin (MacRae and Peterson, 2015). Preliminary behavioral assays involving serotonin and serotonergic psychedelics suggest that fish respond to psilocybin (Braun et al., 2024), though more targeted studies are needed to confirm psilocin formation and distribution in the brain.

For *D. melanogaster*, the situation is less clear. While flies possess phosphatase enzymes that could theoretically mediate the conversion of psilocybin to psilocin (Yang et al., 2000), there is currently no direct evidence showing that this metabolic transformation of psilocybin to psilocin occurs naturally in flies, this remains an open question. However, a recent finding suggests that psilocybin, even without confirmed conversion to psilocin, can exert functional behavioral effects in *Drosophila* (Hibicke and Nichols, 2022), thereby supporting its relevance in neuropsychiatric research, despite unresolved metabolic questions.

**FIGURE 1:**
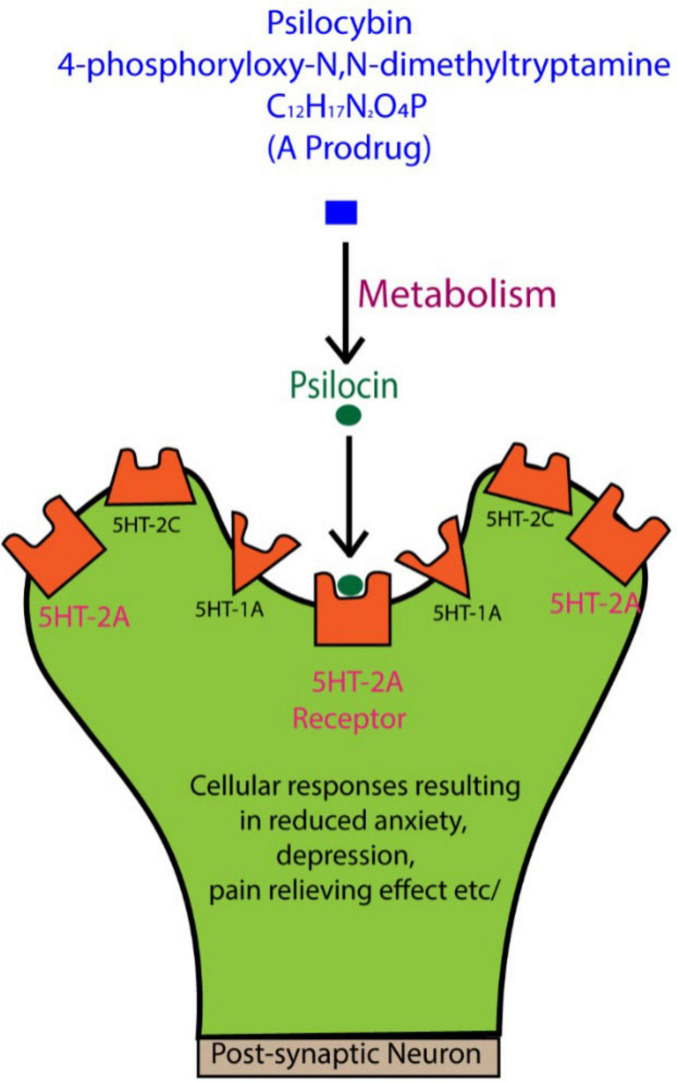
When psilocybin is ingested, it is metabolized to psilocin, which interacts with serotonin (5-HT) receptors, especially the 5-HT2A subtype. These receptors are concentrated in key brain regions, such as the prefrontal cortex, playing a major role in regulating mood, anxiety, and emotional states. While the general effects of psilocin on these receptors are known to influence perception and emotional processing, the detailed molecular mechanisms behind these changes are still not fully understood. Further research is needed to clarify how these interactions contribute to the therapeutic potential of psilocybin for mental health disorders. Figure adapted from Lowe et al. (2021).

Psilocybin is chemically known as 4-phosphoryloxy-*N,N*-dimethyltryptamine, with the molecular formula C_12_H_17_N_2_O_4_P (Nichols et al., 2017), and it is categorized as a tryptamine or indole alkaloid (Plazas and Faraone, 2023). When taken orally in humans, psilocybin is converted in the liver and other tissues into psilocin, the active compound that functions optimally 2–3 h following ingestion and may remain in the body for approximately 6 h, at least in humans (Whelan and Johnson, 2018). Psilocin activates 5-HT receptors, binding strongly to 5-HT2A receptors, and to a lesser degree 5-HT1A and 5-HT2C receptors (Nichols, 2016; Szprȩgiel and Bysiek, 2024; Figure 1).

### 3.2 Psilocybin’s neuropharmacological effects and therapeutic implications

The physiological impact of psilocybin in any animal is not fully understood; however, the few studies addressing mechanistic function have shown that tolerance can be developed across different hallucinogenic substances [e.g., lysergic acid diethylamide (LSD) and psilocybin]. These hallucinogenic substances appear to act through shared pathways, primarily by stimulating 5-HT receptors. This activity not only affects mood and perception but may also influence how animals process pain, potentially altering both pain sensitivity (nociception) and natural pain inhibition (antinociception) (Whelan and Johnson, 2018). One way this pain-relief effect may work is through its interaction with pathways involved in sensing and blocking both neuropathic and musculoskeletal pain (Dworkin et al., 2010; Whelan and Johnson, 2018).

In human studies, psilocybin specifically acts on serotonin receptors (Lowe et al., 2021) and it is considered to have the safest profile among psychedelic drugs (Gable, 2004; Hendricks et al., 2015; Lowe et al., 2021). In humans, the 5-HT2A receptors that process sensory information are located in the thalamus and cortex areas of the mammalian brain. When these 5-HT receptors are activated (Lowe et al., 2021; Teixeira et al., 2022), a reduction in activity in that region appears to occur, resulting in sensory changes often known as hallucinations (Carhart-Harris et al., 2012; Daniel and Haberman, 2017; Nichols, 2004). These receptors are known to influence emotions and moods like anxiety and aggression, as well as cognition, memory, sexual behavior, appetite, and various other biological and neurological processes (Beliveau et al., 2017; Lowe et al., 2021; Nichols and Nichols, 2008).

Psilocybin has also been shown to bind to the 5-HT2A serotonergic receptor subtype with high affinity, while exhibiting poor affinity for the 5-HT1A subtype in rats (Lowe et al., 2021; Passie et al., 2002). In studies on humans using the 5-HT2A antagonist ketanserin, it has been demonstrated that psilocybin and its active metabolite psilocin interact with 5-HT2A receptors to produce psychotomimetic effects. It is also possible that non-5HT2A receptors play a role in mediating psilocybin’s psychopharmacological effects in rodents (Halberstadt and Geyer, 2011) and humans (Lowe et al., 2021).

In the mammalian system, psilocybin stimulates hippocampal and cortical neuroplasticity (Cameron et al., 2021; Nichols, 2004; Raval et al., 2021; Shao et al., 2021), and significantly alters brain dynamics and functional connectivity (Grimm et al., 2018; Lowe et al., 2021; Tagliazucchi et al., 2014) via the breakdown of associative networks as well as the integration of perception networks (Nichols, 2020). Associative networks in the brain connect different regions involved in memory, learning, and higher-order thinking, allowing for complex thought and pattern recognition. Perception networks, on the other hand, process sensory information from the environment, helping to interpret sights, sounds, and other stimuli to create a coherent experience of the world. This separation/breakdown in the networks may act as a mediator between an unrestricted state of cognition and the subjective effects of psilocybin use (Nichols, 2020). Similarly, interactions with feedback loops between the brain and thalamus may be the mechanism of action behind the psychotomimetic effects of psilocybin (Passie et al., 2002). The administration of psilocybin results in widespread cortical activation (Nichols, 2020), and an increased cerebral metabolic rate of glucose breakdown in the putamen, anterior cingulate cortex, and temporal cortex. The neuropharmacological mechanisms of psilocybin are not yet fully understood (de Veen et al., 2017). However, there is evidence that psilocybin interacts not only with the serotonergic system but also, albeit indirectly, with the mesolimbic dopaminergic pathway. The mesolimbic dopaminergic pathway, often referred to as the brain’s reward system, plays a crucial role in processing pleasure and motivation, which is essential for the brain’s reward mechanism. Along the same line, it has also been proposed that in the mesolimbic pathway, dopamine deficit and depression are positively correlated (Dailly et al., 2004; Lowe et al., 2021). Depression is a multifaceted condition that manifests as persistent feelings of sadness, hopelessness, and a lack of interest or pleasure in previously enjoyable activities. As dopamine plays a vital role in mood, motivation, and the brain’s reward system, making it a key factor in understanding psilocybin’s therapeutic potential, a disruption in this pathway, such as reduced dopamine levels, is linked to symptoms like anhedonia (i.e., the inability to feel pleasure), which is common in depression. Although psilocybin primarily affects serotonin receptors, it may also indirectly influence dopamine activity in this reward pathway, potentially restoring balance and improving sensitivity to positive experiences. This dual action on both serotonin and dopamine systems could explain psilocybin’s ability to enhance mood and reduce depressive symptoms, highlighting its unique potential as a treatment for mood disorders. By addressing these interconnected systems, psilocybin offers a promising approach to addressing the underlying causes of depression (Dailly et al., 2004; Lowe et al., 2021).

## 4 Neurophysiological potential and benefits of psilocybin

Psychedelics have the potential to promote the growth of neurons and glial cells, reduce inflammation, and ease oxidative stress, thus making them promising options for treating psychiatric, neurodegenerative, and movement disorders. What sets them apart is their potential to modify the underlying disease by accelerating the recovery process, rather than just alleviating symptoms. Hence, the use of psychedelics as a therapeutic option shows great promise and deserves further development, with a focus on their potential applications in treating neurodegenerative diseases.

As psychedelics have been found to trigger brain processes such as neuroprotection, neurogenesis, and neuroplasticity, protecting neurons and stimulating the growth of new ones, they might be effective in treating major depressive disorders (Flanagan et al., 2019; Ly et al., 2018; Szabó et al., 2021). Research shows that people with depression experience mild brain degeneration with neuron loss and shrinkage in areas like the prefrontal cortex and hippocampus (Manji et al., 2003; Sheline et al., 2003). This degeneration may underlie some of the symptoms associated with depression. Psilocybin and other psychedelics, thought to promote neuroplasticity and neurogenesis, offer potential in targeting these brain changes, especially in the early stages of neurodegenerative diseases. By fostering the growth of neurons and enhancing brain structure, psilocybin could delay symptom onset and support long-term resilience against degeneration in conditions related to both neurodegeneration and mood disorders.

Depression, as earlier defined, is often linked to imbalances in neurotransmitters like serotonin and dopamine, which regulate mood, reward, and stress responses. In animal models, depressive-like behaviors are typically characterized by reduced motivation, impaired decision-making, and diminished social interaction, paralleling human symptoms (Nestler and Hyman, 2010). Anxiety, on the other hand, is a natural response to perceived threats, characterized by heightened alertness, restlessness, and a sense of unease. Biologically, it involves the hyperactivation of the amygdala, a brain region responsible for processing fear, and the dysregulation of neurotransmitters such as serotonin, which help modulate stress responses. In animal models, anxiety-like behaviors are observed through actions such as avoidance of open spaces or bright areas in mazes, mimicking the cautious and hypervigilant states seen in humans (Calhoon and Tye, 2015).

## 5 Case-based perspectives on psilocybin’s clinical promise

### 5.1 Clinical efficacy of psilocybin in treating depression

Recent clinical research has increasingly demonstrated the potential of psilocybin as a fast-acting and durable treatment for depression, particularly in individuals with treatment-resistant or major depressive disorder. Multiple randomized, placebo-controlled trials have shown that a single dose—typically around 25 mg—can lead to rapid and sustained reductions in depressive symptoms when paired with psychological support (Carhart-Harris et al., 2021; Goodwin et al., 2022; Siegel et al., 2024; Raison et al., 2023). Improvements have been observed within days of administration and can persist for several weeks. In addition to symptom relief, some studies have reported enhanced functional outcomes and remission rates, further supporting the therapeutic potential of psilocybin. Collectively, these findings suggest that psilocybin may represent a promising alternative for individuals who do not respond to conventional antidepressant treatments.

### 5.2 Mechanisms of antidepressant action and neural plasticity

Psilocybin treatment has been shown to increase reactivity to positive emotional stimuli in the right amygdala, particularly when combined with psychological support (Carhart-Harris et al., 2017; Roseman et al., 2018). The amygdala is one of the limbic and prefrontal brain regions that is modulated by psilocybin to produce its antidepressant effects (Grimm et al., 2018). The amygdala is a key structure in the brain’s limbic system, which plays a crucial role in processing emotions, particularly fear and reward, and influences emotional learning and memory (LeDoux, 2007). Additionally, psilocybin can reduce or normalize reactivity to stimuli that evoke negative or neutral emotions (DeRubeis et al., 2008; Geyer, 2015).

In studies on healthy volunteers, it was shown that psilocybin reduced the activation of the amygdala in response to visual stimuli associated with threat (Kraehenmann et al., 2015). Specifically, psilocybin decreased the way the amygdala modulates the primary visual cortex in response to threat. Conversely, by reducing the amygdala’s hyper-reactivity to unpleasant emotional cues, some selective serotonin reuptake inhibitors (SSRIs) may produce antidepressant effects by preventing the occurrence of unpleasant feelings. Functional magnetic resonance imaging has been used to examine the effects of psilocybin on cerebral blood flow and blood-oxygen-level-dependent resting-state functional connectivity (Carhart-Harris et al., 2012). An enhanced resting-state functional connectivity within the default-mode network and decreased amygdala cerebral blood flow were linked to fewer depressive symptoms and were reported by the authors after treatment with psilocybin (Roseman et al., 2014).

Psilocybin (at a 25 mg dose) has also been shown in healthy young adults (18–45 years) to interact with neural processes, leading to transformative changes in brain connectivity and activity (Siegel et al., 2024), promoting neuroplasticity, which may help rewire maladaptive neural pathways associated with addiction. These findings highlight psilocybin’s potential as an efficacious treatment option, offering new hope for long-term recovery in individuals struggling with addiction. Neuronal plasticity involves not only growth processes, such as the creation of new neurons and connections, but also the removal of inactive neurons and their connections, suggesting the ability to promote the restoration of the neuronal network and treat neurodegenerative disorders. Future research should focus on exploring the long-term mechanistic and behavioral effects of psilocybin on neuroplasticity with animal models (see below), as well as clinical outcomes in humans. For the latter, conducting large-scale trials to establish optimal dosages and evaluating their impact on cognitive function and emotional regulation across various mental health conditions will be key.

## 6 Models for neurobiological, neurophysiological, and neuropsychopharmacological research

**FIGURE 2:**
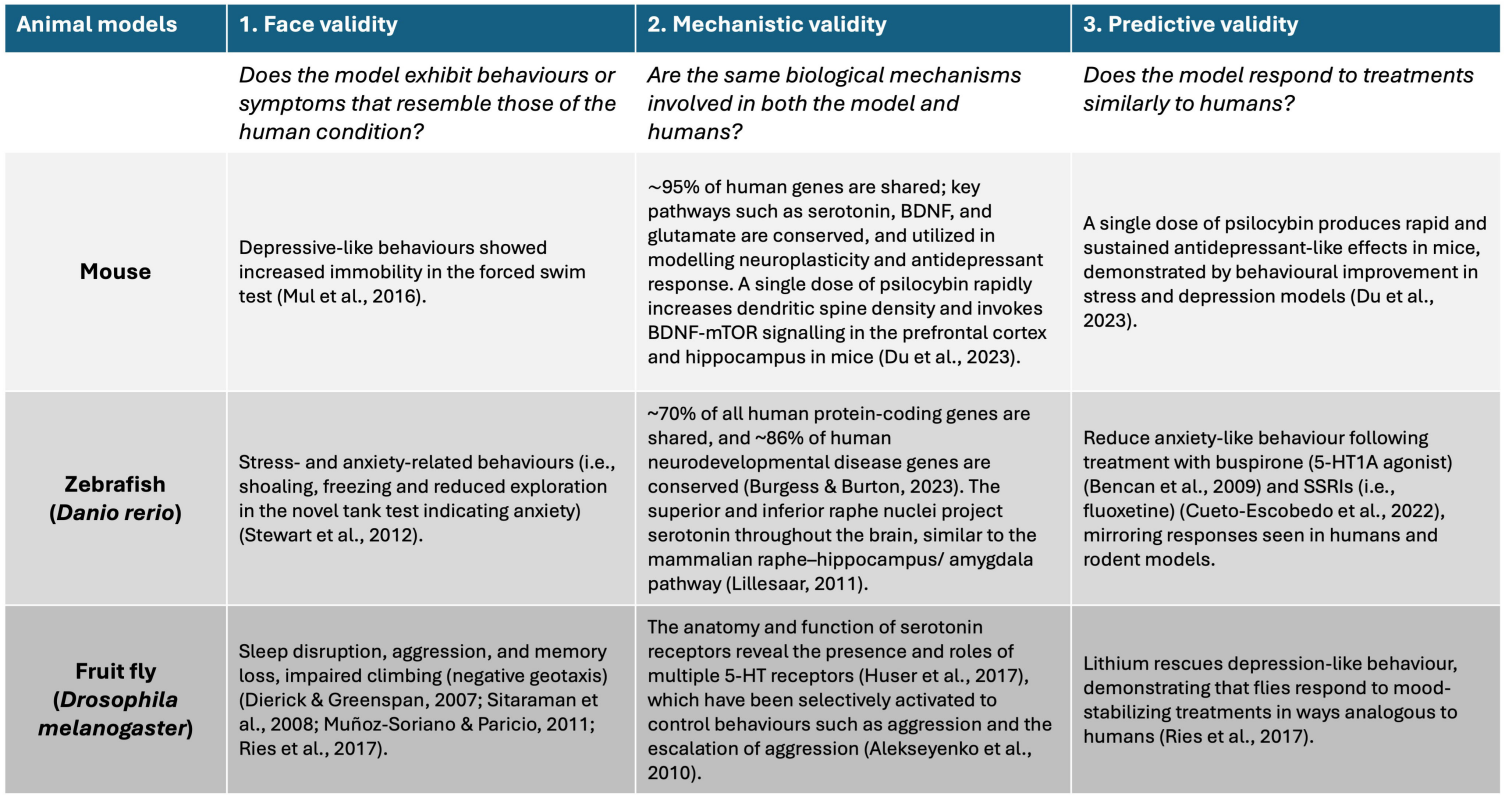
Schematic diagram showing the three types of validity (i.e., face, mechanistic, and predictive) used to assess the suitability of animal models for testing new drugs in relation to human responses. Evidence supporting the use of mouse, fish, and *Drosophila* for each criterion is based on peer-reviewed literature.

Given that the root cause of neuropsychiatric disorders is multifaceted and often involves a significant genetic component, many animal models have been established to aid in the identification and investigation of the molecular and physiological pathways in which these genes play a functional role (Narayanan and Rothenfluh, 2016). To create a valid model of neuropsychiatric disorders in humans, researchers typically focus on three key aspects: (1) Face validity: this refers to whether the model accurately mimics the observable symptoms and behaviors of the disorder. It asks if the model “looks like” the disease, capturing the key features that are characteristic of the human condition. (2) Mechanistic validity: this refers to whether the underlying causes of the disorder in humans, such as genetic mutations or environmental factors, produce similar effects in the model. It is about ensuring that the same biological mechanisms are at play in both the model and the actual disorder. (3) Predictive validity: this assesses whether the model responds to treatments in the same way the human disorder does (Narayanan and Rothenfluh, 2016). A model with high predictive validity can be used to test new drugs or interventions, offering insights into their potential effectiveness in humans (Figure 2). Ideally, a model should meet all three of these criteria, but this is often challenging (Narayanan and Rothenfluh, 2016). Models may only partially capture these aspects due to inherent limitations. Additionally, defining these targets can be problematic because the symptoms, underlying mechanisms, and treatments for many neuropsychiatric disorders are not fully understood (Narayanan and Rothenfluh, 2016). This uncertainty complicates the development of models that are valid across all domains. For example, the symptoms may overlap with those of other conditions, biological markers may be lacking, and effective treatments may be scarce, making it difficult to achieve comprehensive validity (Narayanan and Rothenfluh, 2016). To address these challenges, different animal models such as mouse, fish, and *Drosophila*, offer unique advantages by allowing researchers to investigate various aspects of neuropsychiatric disorders.

### 6.1 Mouse as model

The use of the mouse as a model in neurobiological, neurophysiological, and neuropsychopharmacology research on psilocybin has provided valuable insights into both the molecular and behavioral effects of psychedelics. Mice are favored due to their genetic similarity to humans, well-characterized neural circuits, and the availability of transgenic lines that allow precise manipulation of brain regions. These features are crucial for understanding the neurobiological underpinnings of psilocybin’s therapeutic potential.

Not all animal behavior models can fully reflect the changes in perception, thinking, and mood that hallucinogens cause in humans; however, rodents may show behaviors that resemble the effects of hallucinogens in humans (González-Maeso et al., 2007). When hallucinogens (including psilocybin) are given to rats and non-human primates, they cause several natural reactions, including changes in their exploratory behaviors (Adams and Geyer, 1985; Hibicke et al., 2020; Zhao et al., 2024), hyperthermia, twitching of the head (Halberstadt et al., 2020), scratching of the ear (Corne and Pickering, 1967; Darmani et al., 1990). These changes in behavior can be used to target certain psychological and stress-induced effects and to monitor the activity of the drug. Behavioral experiments involving psilocybin-treated mice have demonstrated its ability to enhance neuroplasticity (Zhao et al., 2024), modulate stress responses (Zhao et al., 2024), and improve learning and memory processes (Barrett et al., 2020a; Zhao et al., 2024). These effects have implications for treating disorders such as depression, anxiety, and PTSD (Duman et al., 2016; Zhao et al., 2024).

Using the mouse as a model in studies involving psilocybin and other psychoactive substances offers several advantages. One significant benefit is their genetic similarity to humans (Swanzey et al., 2021); (Nadeau and Auwerx, 2019) which allows researchers to investigate how specific genes influence brain function (Nadeau and Auwerx, 2019) and behavior (Bućan and Abel, 2002). In addition, the genome of the mouse can be edited using the CRISPR/Cas9 technique (Cong et al., 2013; Jinek et al., 2012). With the development of transgenic mice (Nadeau and Auwerx, 2019; Swanzey et al., 2021), scientists can therefore manipulate genes of interest in mouse (Long et al., 2014) and then use the methodology and technique to understand how molecular pathways, like serotonin receptor systems, respond to psychedelics. This genetic control helps create models of psychiatric conditions such as depression or anxiety, making it easier to study the potential therapeutic effects of psilocybin in a controlled, reproducible manner as done recently (Hesselgrave et al., 2021).

In mice, psilocybin has been reported to have the potential to promote neuroplasticity (Kobayashi et al., 2010; Sale et al., 2014; Vetencourt et al., 2008), which is considered an important aspect of its therapeutic impact (Josephy-Hernandez et al., 2017; Kobayashi et al., 2010; Sale et al., 2014; Shao et al., 2021; Vetencourt et al., 2008). Additionally, it has been shown that psilocybin affects the mouse’s functional connectivity throughout the entire brain region (Grandjean et al., 2021). This neuronal plasticity is made possible by the brain-derived neurotrophic factor and its receptor, the neurotrophic receptor tyrosine kinase, Ntrk2 (Castrén and Antila, 2017). Collectively, these signaling components play a crucial role in the effectiveness of psilocybin (Carhart-Harris et al., 2016; Castrén and Antila, 2017; Davis et al., 2021; Griffiths et al., 2016).

It has been suggested that antidepressants result in plasticity by promoting synaptogenesis, increasing the rate at which connections between neurons are renewed in rats (Bessa et al., 2009; Castrén and Antila, 2017; Hajszan et al., 2009) and mice (Castrén and Antila, 2017; Chen et al., 2011). This antidepressant activity has also been reported to promote axonal and dendritic remodeling in the hippocampus (Castrén and Antila, 2017; Sairanen et al., 2007; Wang et al., 2008) and the prefrontal cortex in Wistar rats (Bessa et al., 2009; Castrén and Antila, 2017). These findings highlight psilocybin’s promising potential for promoting neuroplasticity as a therapeutic strategy for mood disorders, a concept that aligns with the growing interest in using psychedelics.

The relatively short lifespan and fast breeding cycle of mice (Long et al., 2022) allow researchers to conduct longitudinal studies efficiently. This is especially useful when examining the long-term impact of psilocybin on brain plasticity, neurogenesis, or behavior over multiple generations (Zhao et al., 2024). Mice are also suitable for behavioral assays—such as the open-field test or fear-conditioning protocols (Zhao et al., 2024) —that mimic key aspects of human mental health conditions (Hesselgrave et al., 2021). These advantages position the mouse as an essential tool for advancing therapeutic research on psychedelics and brain function.

Mouse studies have been pivotal in revealing how psilocybin promotes neuroplasticity. For instance, a single dose of psilocybin has been shown to increase expression of plasticity-related genes (such as *c-Fos*, *BDNF*, and *mTOR*) and enhances dendritic spine density and complexity in both the hippocampus and frontal cortex, these are effects that are detectable even a week later (Du et al., 2023; Weiss et al., 2025). These structural and genetic changes suggest a strengthened capacity for learning, memory, and stress resilience, which are key factors in psilocybin’s antidepressant and anti-PTSD potential.

Additionally, mice treated with psilocybin during fear-conditioning studies exhibit accelerated fear extinction, a behavioral indication that these neurobiological changes are functionally protective. The treatment rescues deficits in hippocampal structure (e.g., spine density), protein expression (BDNF, mTOR), and neurogenesis markers (DCX, BrdU) while simultaneously reducing fear-related behavior (Du et al., 2023). This demonstrates psilocybin’s dual role in enhancing brain resilience and improving behavioral outcomes.

Collectively, these findings, supported by mouse genetic models and transgenic tools, firmly link molecular and structural brain changes to functional and neuroprotective effects. They provide strong preclinical evidence that psilocybin induces lasting neural repair and adaptation, thus justifying its exploration in clinical trials for mood disorders, PTSD, and neurodegenerative diseases. Also, psilocybin’s ability to induce neurogenesis and synaptic remodeling could provide a novel approach to treating conditions like depression and anxiety, marking a shift from traditional treatments toward more transformative interventions.

### 6.2 Fish as a model

Fish models are valuable in neuroscience research because their complex nervous systems share both structural and functional similarities with those of humans (Matsui, 2017). As vertebrates, fish possess highly conserved genes and essential biological pathways that are similar to those in humans. Notably, fish share approximately 70% of human genes, and approximately 84% of known genes have a human disease-related ortholog (Don et al., 2024; Kodera and Matsui, 2022). Their comparable neuronal structures and functions, including the same cell types as humans, further establish them as excellent models for neuroscience research (Don et al., 2024; Northcutt, 2002). Several fish species, such as zebrafish (*D. rerio*), African turquoise killifish (*Nothobranchius furzeri*), medaka (*Oryzias latipes*), and mangrove rivulus (*Kryptolebias marmoratus*), offer diverse research opportunities depending on the specific questions posed by the researchers.

Fish have unique features that provide advantages over mammalian models. Most fish undergo external fertilization, and their transparent embryos and larvae allow for remarkable visibility during neuronal development (Kodera and Matsui, 2022). This level of observation is difficult in mammalian models as their fertilization occurs internally. Additionally, most fish have a relatively short development period and often a shorter life cycle compared to mammals, which facilitates studies on genetic modifications and development processes (Matsui, 2017).

Zebrafish stand out as advantageous models due to their ease of genetic manipulation with the CRISPR/Cas9 (Varshney et al., 2015). This method enables researchers to create specific mutations that can reveal the roles of particular genes, resulting in the generation of transgenic or knockout lines (Choi et al., 2021). These transgenic fish allow researchers to label specific populations of neurons, facilitating the study of neuronal networks and their relationship to behavior (Choi et al., 2021; Varshney et al., 2015). Fish facilitate studies of environmental and genetic interactions and their influence on behavior (Kelley et al., 2016). They exhibit complex behaviors (e.g., social, reproductive, territorial) and neuronal mechanisms, making them excellent models for behavioral experiments in neuroscience. Behavioral experiments in zebrafish have further demonstrated psilocybin’s potential to reduce anxiety-like responses and encourage adaptive coping under stress (Braun et al., 2024).

Many fish have well-established assays for assessing anxiety, stress, and learning behaviors, along with many others (Hamilton et al., 2021; Rossi and Wright, 2021; Stewart et al., 2012). For example, zebrafish are known for their shoaling behavior (Miller and Gerlai, 2011), while other fish species are typically solitary, allowing for the assessment of social interactions, including aggression levels (Taylor, 1990; Taylor et al., 2008). This behavioral diversity across species is important when examining specific neurological diseases. Given the similarities in neuronal pathways, including transmitters, receptors, and hormones (Panula et al., 2006), fish models can help reveal human neurological processes. Due to the numerous neurological pathways conserved between fish and humans, experiments using fish models enable researchers to investigate neurodegenerative diseases and conduct pharmacological testing for *in vivo* treatments. Neurological diseases such as Down syndrome, autism, schizophrenia, and intellectual disabilities can be modeled in fish (Don et al., 2024).

Regarding brain structures, in the teleost or bony fish, two raphe nuclei (the superior raphe and inferior raphe) connect to most areas of the brain, like the mammalian system (Lillesaar, 2011). Additionally, like in other fish, many areas of the telencephalon receive moderate to extensive serotonergic input from the raphe. This includes regions such as the lateral and medial zones of the dorsal telencephalon, which are functionally similar to the mammalian hippocampus and amygdala, respectively (Lillesaar, 2011; Puglisi-Allegra and Andolina, 2015). Fish and other vertebrates show similarities not just in their anatomy, but also in their genes and behaviors. However, there are also apparent differences; thus, more research is needed to understand which genes, enzymes, transcription factors, and regulatory networks are shared, and how they contribute to adaptive behavior in fishes and other vertebrates (Puglisi-Allegra and Andolina, 2015).

Fish models have become valuable tools for studying how psilocybin affects the brain. Their genetic and neurological similarities to humans (Don et al., 2024; Choi et al., 2021), make them well-suited for examining the molecular basis of neuroplasticity and neuroprotection (Kodera and Matsui, 2022). Transparent embryos and fast development allow researchers to observe brain changes in real-time (Choi et al., 2021; White et al., 2008), including how brain circuits respond to drugs as pharmacological interventions (Chia et al., 2022). Genetically modified zebrafish, such as those created using CRISPR/Cas9, help identify specific genes and neural pathways involved in the brain’s response to drugs as pharmaceutical interventions (Choi et al., 2021). Furthermore, the fish model has facilitated detailed investigation of serotonin pathways, particularly the raphe nuclei and their projections to brain regions comparable to the mammalian hippocampus and amygdala, which are areas that play key roles in emotional regulation and memory (Lillesaar, 2011; Panula et al., 2006). Psilocybin’s interaction with these serotonergic systems provides insight into how it may promote brain flexibility and recovery. These findings, along with the ability to measure brain activity and molecular responses, position fish as a promising model for uncovering how psychedelics might support brain repair and resilience, especially in the context of mood disorders and neurodevelopmental conditions.

### 6.3 *Drosophila* as a model

The seemingly simple fruit fly (*Drosophila melanogaster*) is a valuable model for studying neuropsychiatric disorders due to the many fundamental biological, physiological, and neurological processes that are shared between *Drosophila* and mammals (Edgar and Lehner, 1996; Rubin et al., 2000). This became apparent in the 1980s when researchers discovered developmental genes in *Drosophila* that dictate its body plan. Despite the significant differences in morphology between *Drosophila* and mammals, the genes involved in these developmental processes are highly conserved across species (Gehring et al., 2009; Narayanan and Rothenfluh, 2016), and it is even possible to generate fully formed ectopic fly eyes by misexpressing a developmental gene from the mammalian eye into *Drosophila* (Halder et al., 1995; Narayanan and Rothenfluh, 2016).

*Drosophila* has a short generation time of less than two weeks, it produces vast numbers of genetically identical offspring, and it is inexpensive and simple to maintain in the laboratory. These characteristics make the fruit fly ideal for forward genetic screens, which involve testing large populations of genetically heterogeneous flies for specific phenotypes, thereby identifying and studying the mutant genes that cause them. The use of *Drosophila* as a disease model has been essential for gaining a fundamental understanding of certain important processes, despite some limitations due to anatomical differences between humans, mammals, and flies. In *Drosophila*, chemical screens have been effectively carried out for many disorders (both developmental and non-developmental), involving the kidney, metabolism, and central nervous system (Gasque et al., 2013; Hofherr et al., 2016; Mirzoyan et al., 2019; Whitworth et al., 2006). Therapeutics have greatly benefited from the detailed characterization of JAK-STAT, APC, Wnt, Notch, and other signaling molecules that are shared by humans and *Drosophila*. Pharmacological investigations against multiple drugs are achievable due to the availability of *Drosophila* models. Furthermore, biomedical disorders can be modeled in the *Drosophila* system, making it of immense potential for personalized medicine.

Transgenic *Drosophila* strains can be used to study neurodevelopmental and neurophysiological disorders, and this can be achieved via the GAL4/UAS system. This GAL4/UAS technique uses a promoter that contains upstream activating sequences (UAS) and the transcription factor GAL4, which is derived from yeast. It functions as a transactivator that can be controlled by a promoter or enhancer trap (Brand and Perrimon, 1993). In the end, when flies carrying the Enhancer-GAL4 are crossed with flies carrying the UAS-target gene, this results in progeny with the induced target gene (Brand and Perrimon, 1993).

**FIGURE 3:**
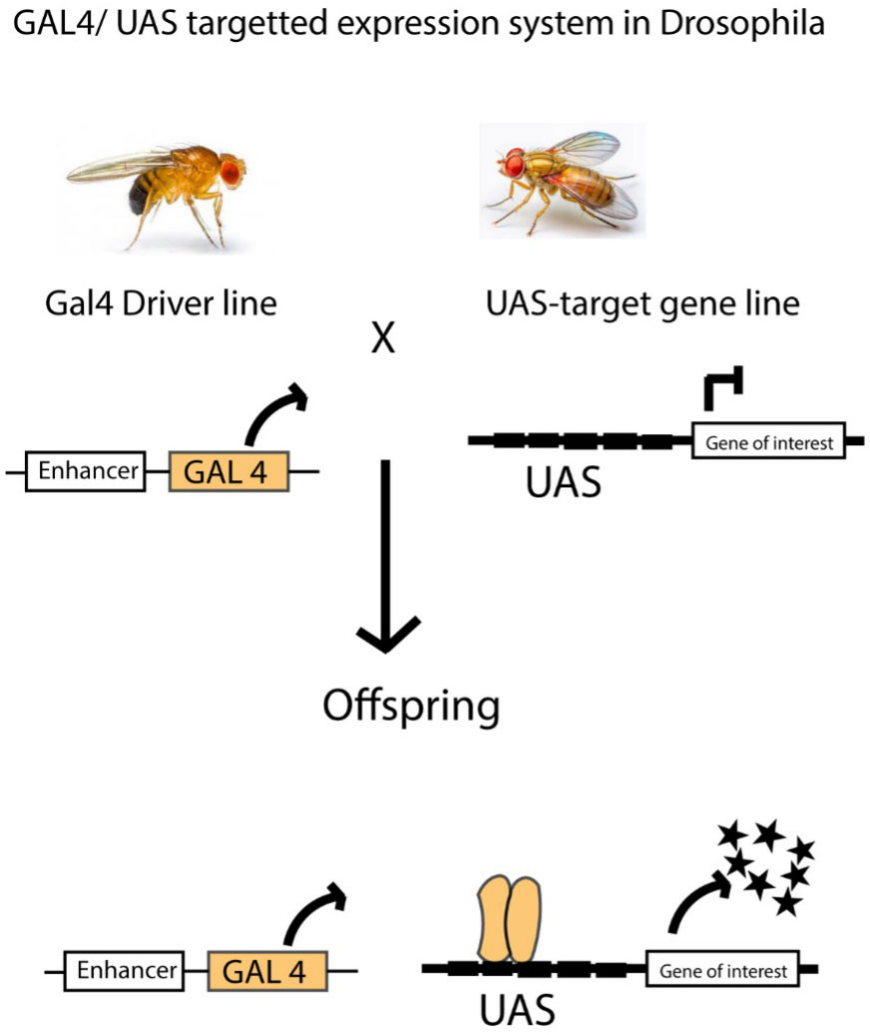
In *Drosophila*, the GAL4/UAS system is widely used to control gene expression in specific tissues. This method works by linking the expression of the GAL4 transcription factor to a tissue-specific enhancer. In a separate line of flies, the gene of interest is placed under the control of a UAS (Upstream Activation Sequence). When these two lines are crossed, the offspring express the target gene only in cells where the GAL4 transcription factor is active. This allows for precise spatial and temporal control of gene expression. The GAL4/UAS system is incredibly versatile and has been applied in many experiments. It can be used to label specific cells, activate or silence genes, and even study protein-DNA interactions on a genome-wide scale. This flexibility makes it a powerful tool for studying gene function, neural circuits, and developmental processes in a controlled and targeted way. Figure adapted from Caygill and Brand (2016).

With the help of the binary Gal4/UAS system in the *Drosophila* model (Alekseyenko et al., 2010; Brand and Perrimon, 1993), we can either introduce an immediate or long-lasting modulation of amine neuronal functions at various stages of development and in adulthood. Hence, activating the 5-HT2A receptor via the GAL4/UAS system makes the neuron more responsive to signals, thereby increasing its excitability (Teixeira et al., 2022). So far, both pharmacological and genetic techniques have been used to propose potential roles for 5-HT neurons in sleep (Yuan et al., 2006), memory (Sitaraman et al., 2008), and aggression (Dierick and Greenspan, 2007) in *Drosophila*. While studies have shown that psilocybin affects serotonin receptors and alters brain dynamics in both mammals and humans, there is still much to uncover about the precise mechanisms underlying these effects. The long-term impact on neuroplasticity, and the differences across species remain open questions. In *Drosophila*, the use of the Gal4/UAS system provides a powerful tool for investigating serotonin receptor function; however, further research is needed to fully understand how psilocybin modulates neuronal activity and behavior in simpler animal models (Figure 3).

The use of *Drosophila* as an animal model for neurobiological research may accelerate discovery by quickly identifying promising active ingredients for further evaluation and assessment. It is a more efficient screening tool than the *in vitro* cell culture approach for reducing the number of possible treatments to test before investing in more costly mammalian models. While *Drosophila* models are valuable in the discovery phase, efficiency and success require a clear hypothesis and a strong understanding of the limitations of the *Drosophila* model.

It is noteworthy that *Drosophila* shares many conserved genetic, neurochemical, and developmental pathways with humans (Narayanan and Rothenfluh, 2016; Rubin et al., 2000). These similarities have made the fly an effective system for dissecting basic principles of brain function and neuroplasticity *in vivo*. One of the key advantages of using *Drosophila* lies in its genetic accessibility. Through well-established tools like the GAL4/UAS binary expression system (Brand and Perrimon, 1993), via which researchers can selectively activate or suppress genes in specific neurons, including 5-HT producing ones.

While no current studies directly examine psilocybin’s effects on neuroplasticity or neuroprotection in *Drosophila melanogaster*, the model remains a powerful genetic tool for investigating serotonergic pathways and neuroactive compounds. Given its well-mapped nervous system and genetic tractability, it is possible to examine psilocybin’s interaction with serotonin receptors, particularly 5-HT2A receptors, which are known targets of psilocybin, and assess how these interactions influence behavior, neuronal excitability, neuroplasticity, and neuroprotection in flies. Future research using *Drosophila* could provide critical insights into the cellular and molecular changes induced by psilocybin, particularly through manipulation of 5-HT2A receptor circuits and behavioral assays (Teixeira et al., 2022).

## 7 Considerations when using animal models

The use of animals in drug discovery and development is limited by the evolutionary differences between these models and humans. *Drosophila* and other small model organisms are useful for rapidly analyzing the *in vivo* mechanism of action of various active compounds, while mammalian models offer the affordability and suitability that are essential for defining critical pharmacokinetic factors like absorption, distribution, and metabolism in the subsequent phase of drug development (Mirzoyan et al., 2019) which are limitations in the small model organisms. When using non-mammalian models, it is important to consider the direct and indirect effects of psilocybin on the immune system. Mammals are known to have a complex adaptive and innate immune system, but *Drosophila* has a relatively simple innate immune system. Furthermore, *Drosophila* lacks all the cell types found in mammalian neural systems, and the *Drosophila* brain is notably less structurally and functionally advanced compared to mammalian models (Adeyinka and Egger, 2023).

Fish models are generally more advanced compared to *Drosophila* in terms of immune system complexity, as they exhibit both innate and adaptive immune responses (Zhang et al., 2024). Fish possess the same cell types as humans in their neural systems, but they differ in their structure and complexity (Don et al., 2024). That said, there is a notable gap in therapeutic research focusing on the effect of psilocybin on fish species. While studies have explored the effects of other similar hallucinogens such as LSD, 3,4-methylenedioxymethamphetamine (MDMA), and mescaline (Grossman et al., 2010; Kyzar et al., 2012; Stewart et al., 2011; Green et al., 2012; Hagen et al., 2024), research specifically addressing psilocybin remains limited. This gap presents an opportunity for further investigation into how psilocybin and other hallucinogens may impact neurobiological processes and behavior in fishes, potentially shedding light on their effects in mammals.

One of the key challenges in psychedelic research, particularly with psilocybin, is the difficulty of accurately modeling complex psychiatric conditions in animals. While animal models, especially rodents, can mimic some isolated behaviors linked to psilocybin use in humans, they fall short in capturing the full range of human responses, especially those involving subjective experiences, mood, and perception (Hanks and González-Maeso, 2013). For example, rodent behaviors like the head-twitch response are commonly used to indicate activation of the serotonin 5-HT2A receptor, the main target of psilocybin. Other behaviors, such as altered locomotion, disrupted startle reflex (prepulse inhibition), and changes in exploratory activity, help researchers identify general psychoactive effects. However, these behaviors do not fully reflect the psychological or emotional dimensions of psilocybin’s impact on humans (Hanks and González-Maeso, 2013).

While such models are useful for understanding some of the basic molecular and neural pathways involved, they cannot account for higher-order human experiences such as ego dissolution, mystical insights, or therapeutic breakthroughs. This limitation complicates the interpretation of findings from animal studies and their application to clinical contexts (Hanks and González-Maeso, 2013). Additionally, most animal models lack essential components present in human psychedelic therapy, such as verbal preparation, therapeutic relationship, and expectancy, which significantly affect clinical outcomes. Therefore, as animal models remain important for uncovering how psilocybin works at the receptor and circuit level, their results must be interpreted cautiously when predicting effects in humans. Bridging this gap requires a careful balance between preclinical insights and findings from well-designed human trials.

Another major challenge in translating psilocybin findings from animals to humans is the marked difference in how each species metabolizes the compound. It has been reported that psilocybin enhances glucose metabolism and glutamate signaling in humans over the course of its approximate 6-h half-life (Gouzoulis-Mayfrank et al., 1999; Mason et al., 2020; Vollenweider et al., 1997).

Psilocybin acts as a prodrug, swiftly converting into its active form, psilocin, via dephosphorylation in both animals and humans (Lowe et al., 2021; Thomann et al., 2024; Whelan and Johnson, 2018). However, the pharmacokinetic timelines vary substantially across species: in humans, psilocin plasma levels peak approximately two hours after oral administration and have a half-life of 2–3 h (Griffiths et al., 2016; Johnson et al., 2017; Thomann et al., 2024), whereas in mice, psilocin is cleared more rapidly, often with a half-life of less than one hour (Holze et al., 2023; Thomann et al., 2024). The metabolic timeline of psilocybin in *Drosophila* and fish remains poorly understood, highlighting an important area for future research (Thaoboonruang et al., 2024). These interspecies differences in absorption and elimination not only impact the duration of subjective effects but also complicate dosage normalization when translating from animal models to clinical settings (Thomann et al., 2024).

In addition to kinetic differences, the metabolic pathways of psilocin exhibit species-specific variations that further complicate interpretation. In humans, psilocin undergoes extensive glucuronidation and is excreted as psilocin-O-glucuronide and 4-HIAA, with only about 1–3% excreted unchanged (Thomann et al., 2024). Rodents, by contrast, may rely more heavily on oxidative and MAO-mediated deamination pathways, which may generate distinct metabolites affecting receptor activation and systemic exposure (Thomann et al., 2024). The result is that equivalent doses in animal models might not produce comparable brain concentrations or receptor engagement to those seen in humans, complicating the translation of efficacy and safety profiles.

Ultimately, these pharmacokinetic and metabolic differences must be taken into account when interpreting behavioral assays in preclinical research. Hence, we suggest that accurate cross-species comparisons require careful pharmacokinetic modeling and consideration of metabolic fate, thus ensuring that behavioral findings in rodents remain meaningfully predictive of clinical outcomes.

## 8 Furthering psilocybin’s therapeutic potential

To fully realize the therapeutic potential of psilocybin, future research should focus on several critical areas. One key priority is optimizing dosage. While early clinical trials have shown that a single or two-session dose can provide lasting improvements in depression, more research is needed to determine the safest and most effective dosing schedules for different individuals and conditions (Carhart-Harris et al., 2021; Carhart-Harris et al., 2021; Davis et al., 2021) which would help in the understanding of how often psilocybin can be used without losing its therapeutic benefit or causing harm, which is essential for guiding long-term treatment strategies.

Another important area for future research is identifying biological markers, also known as biomarkers, that can help predict an individual’s response to psilocybin. These could be brain imaging markers to track changes in brain activity, blood markers to track blood-based inflammatory markers, stress hormone levels, genetic traits/factors or even features of the psychedelic experience itself. For example, a recent systematic review found that people who had a stronger emotional or mystical experience during their psilocybin session were more likely to show lasting improvements in mental health symptoms (Ko et al., 2022). This suggests that the quality of the experience may be an early indicator of who benefits most.

One promising brain-based biomarker candidate is SV2A, a protein associated with synaptic density, which indicates the extent to which brain cells are connecting and communicating. Studies in animal models have shown that psilocybin can increase SV2A levels, and new imaging techniques in humans, such as PET scans, may make it possible to track these changes over time (Olson, 2025). We propose that this could make psilocybin treatment more targeted, predictable, and effective, offering a more precise way to measure psilocybin’s effects on the brain and help tailor treatments to individual needs.

Future research may focus on examining how psilocybin interacts with systems beyond the brain, such as the immune system, stress-response pathways, and the gut–brain axis. For example, a well-controlled study in healthy volunteers found that a single moderate dose of psilocybin caused measurable changes in immune markers that lasted at least a week and even reduced cortisol responses following a stress challenge (Mason et al., 2023). This suggests that psilocybin could support stress resilience and modulate inflammation, both of which are key factors in mood and neuropsychiatric disorders. Examining the gut–brain axis, the gut microbiome affects stress hormones, immune responses, and brain function, all of which are crucial to mental health. Recent reviews by Kelly et al. (2023), and also Caspani et al. (2024), tell us that psychedelics, like psilocybin, may temporarily alter gut microbiota or gut-brain signaling, potentially enhancing therapeutic effects during treatment phases and integration periods.

As techniques such as transcriptomics, CRISPR-based gene editing, and circuit-level mapping in animals now make it possible to dissect which genes and pathways are critical for psilocybin’s effects (Jefsen et al., 2021), research can now focus on those involved in mood regulation, neural plasticity, and immune signaling, thus revealing mechanisms that could be targeted in human treatment. These tools would enable researchers to explore not only the brain, but also how psilocybin affects whole-body systems in a controlled, mechanistic manner, thereby laying the foundation for innovative treatments for mood disorders and neurodegenerative diseases such as Alzheimer’s and Parkinson’s, where cross-talk between the brain and peripheral systems occurs.

## 9 Conclusion

As neurodegenerative diseases pose a significant and increasing challenge for modern societies, and the progress in developing effective treatments has fallen behind compared to fields like cardiovascular disease and cancer, the use of psilocybin as an emerging therapeutic option in humans may become a suitable treatment to help reverse these troubling neurological trends.

Non-mammalian models, such as *Drosophila* and fish, have emerged as valuable systems for advancing our understanding of psilocybin’s mechanisms of action and an effective possible treatment option for neurodegenerative diseases and mental health disorders. Therapies with psychedelic assistance via these models can offer fresh perspectives on pressing problems with the existing standard of care for mental illnesses. Decades of multiple clinical investigations and thousands of years of anecdotal tales have suggested that psilocybin-assisted treatment may be practical, efficacious, safe from a toxicological standpoint, and physiologically effective (Gable, 2004). It may also have potential in the treatment of mental disorders (Lowe et al., 2021; Mahapatra and Gupta, 2017; Passie et al., 2002).

Psilocybin is currently being suggested as a potential alternative for treating neurodegenerative disorders in humans, as the possibility of an adverse effect is relatively low, and non-addictive, as they do not lead to compulsive drug-seeking behavior, and do not cause withdrawal symptoms (Johnson et al., 2008). Considering some neurological disorders, such as amnestic mild cognitive impairment, with no current effective clinical medications available, psilocybin might offer some hope.

Looking ahead, to fully realize the therapeutic potential of psilocybin, it is essential to advance research in both clinical and preclinical settings, with a particular emphasis on understanding its effects at the molecular, cellular, and systemic levels. Small animal models such as *Drosophila* and fish, which have emerged as valuable tools for studying the neurobiology of psilocybin, should remain a cornerstone of this effort. These models not only provide insights into fundamental mechanisms such as neuroplasticity, synaptogenesis, and neurotransmitter dynamics but also enable high-throughput genetic and pharmacological screening that would be impractical in mammalian systems. A focused direction for future research involves leveraging these models to elucidate the pathways through which psilocybin modulates neural circuits, behavior, and resilience against neurodegenerative processes. This knowledge could then inform targeted interventions and enhance the translational relevance of findings to mammalian and human studies.

Future efforts should also prioritize the development of advanced methodologies to study the long-term impact of psilocybin on brain structure and function. Investigating its potential as a disease-modifying agent in disorders like Alzheimer’s, Parkinson’s, and depression, rather than merely alleviating symptoms that could redefine therapeutic strategies.

On a broader scale, fostering interdisciplinary collaborations among neurobiology, psychiatry, and pharmacology will be crucial for translating findings from small animal models into meaningful clinical applications. Large-scale studies combining molecular insights from *Drosophila* and fish with data from rodent models and human clinical trials could accelerate progress in this field. Moreover, we are proposing that a regulatory framework should evolve to accommodate the unique nature of psychedelics, ensuring safe and ethical use while encouraging innovation. By addressing these challenges and opportunities, research on psilocybin can pave the way for transformative advancements in the treatment of neurodegenerative diseases, mood disorders, and beyond.
